# Comparison of the Medium‐term Outcomes of Anterior Lumbar Discectomy and Fusion with Minimally Invasive Transforaminal Lumbar Interbody Fusion: A Retrospective Cohort Study

**DOI:** 10.1111/os.14028

**Published:** 2024-03-26

**Authors:** Chao Song, Zhibo Deng, Hanhao Dai, Wu Zheng, Guoyu Yu, Yijing Wu, Jun Luo, Jie Xu

**Affiliations:** ^1^ Department of Orthopedics Shengli Clinical Medical College of Fujian Medical University Fuzhou China; ^2^ Department of Orthopedics Fujian Clinical Research Center for Spinal Nerve and Joint Diseases Fuzhou China

**Keywords:** Anterior lumbar discectomy and fusion, LDDs, Microscope, Minimally invasive transforaminal lumbar interbody fusion

## Abstract

**Objective:**

Lumbar degenerative diseases (LDDs) with huge herniation in the left lateral recess or central canal present challenges for oblique lateral lumbar interbody fusion (OLIF) or endoscope‐assisted OLIF procedures. Currently, minimally invasive transforaminal lumbar interbody fusion (MIS‐TLIF) is the primary approach for this issue. This study aims to provide a standardized technical description of the anterior lumbar discectomy and fusion (ALDF) and evaluate the medium‐term clinical effectiveness of both ALDF and MIS‐TLIF techniques.

**Methods:**

A retrospective review was performed on LDDs who underwent ALDF and MIS‐TLIF surgery from January 2018 to January 2020. The evaluation encompassed various clinical outcomes, such as the visual analogue scale (VAS) scores for back pain and leg pain (VAS‐back, VAS‐leg), the Oswestry disability index (ODI), the 36‐item short‐form health survey mental component summary (SF‐36 MCS), and the physical component summary (SF‐36 PCS). Additionally, radiological parameters, including disc height (DH), segmental disk angle (SDA), lumbar lordosis (LL), and cross‐sectional area (CSA), were assessed. Data including radiculopathy, estimated blood loss, operation time, time of getting out of bed, fusion rate, and complications were recorded. Student's independent samples *t* test and Pearson's chi‐square test were used to compare the differences between groups.

**Results:**

In total, 47 patients were treated by ALDF and 48 patients were treated by MIS‐TLIF. The ALDF group exhibited statistically significant lower estimated blood loss and earlier time of getting out of bed compared to the MIS‐TLIF group (*p* < 0.05). The ALDF group demonstrated lower VAS‐back scores and a higher remission rate of low back pain 3 years after the surgery (*p* < 0.05). During the entire follow‐up period, the ALDF group exhibited higher increases in DH and SDA compared to the MIS‐TLIF group (*p* < 0.05). At 6 months, the fusion rate in the ALDF group was significantly higher than in the MIS‐TLIF group (*p* < 0.05). The comparison revealed no statistically significant differences in complication rates between the two groups (*p* > 0.05).

**Conclusion:**

The ALDF could be considered as a viable surgical alternative for the treatment of LDDs that necessitate ventral neural direct decompression. ALDF exhibited favorable medium‐term outcomes in patients with LDDs, displaying advantages in facilitating expedited recovery, enhancing radiographic outcomes, and elevating the remission rate of low back pain. Although ALDF presents slightly higher complication rates compared to MIS‐TLIF, it does not adversely affect clinical outcomes.

## Graphical abstract

Illustration of the specially designed rotary distractor. (a–c) The operation processes of the rotary distractor in ALDF surgery. (d) The disc fragment was carefully removed.

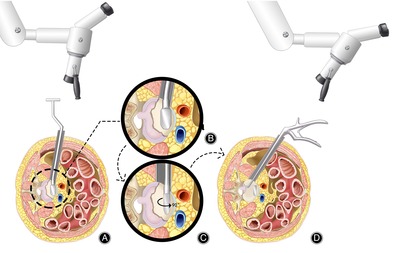

## Introduction

As minimally invasive surgical procedures for treating lumbar degenerative diseases (LDDs), oblique lateral lumbar interbody fusion (OLIF) and minimally invasive transforaminal lumbar interbody fusion (MIS‐TLIF) have gained wide acceptance.^1^, ^2^, ^3^, ^4^ OLIF is generally deemed unsuitable for LDDs with huge disc herniation, attributed to its limited indications in cases of concurrent ruptured or extra‐ligamentous disc herniation.^5^, ^6^, ^7^ Shin *et al*. reported that microscope‐assisted anterior lumbar interbody fusion (ALIF) shows an excellent surgical outcome in foraminal stenosis caused by foraminal sequestrated disc herniation.^8^ However, it is unsuitable for coexisting central canal or lateral recess stenosis, as well as completely collapsed disc space.^8^ Various studies have demonstrated that spinal endoscope‐assisted OLIF results in successful direct neural decompression in degenerated lumbar diseases with huge lumbar disc herniation or cauda equina syndrome.^6^, ^9^, ^10^, ^11^ The constraints associated with spinal endoscopic discectomy‐assisted OLIF, particularly concerning pathologies in the left foramen, have been elaborated.^6^ The advanced oblique lateral endoscopic decompression and interbody fusion (OLEDIF) technique facilitates precise visualization of the posterior annulus fibrosus, nucleus pulposus, and osteophytes, achieved through a 30°–60° lens end view. However, its applicability is predominantly restricted to cases involving large herniated or prolapsed discs situated in the right lateral recess or central canal.^11^ Meanwhile, endoscopic lumbar interbody fusion remains challenging for patients with bilateral foraminal stenosis, severe central stenosis, or high‐grade spondylolisthesis.^12^

For different types of LDDs, the indications and contraindications should be comprehensively considered to select the appropriate lumbar fusion surgery. MIS‐TLIF is considered an option when a herniation is associated with evidence of spinal instability, chronic low back pain, bi‐radicular symptoms, and/or severe degenerative changes.^13^ In MIS‐TLIF, the establishment of the trans‐multifidus decompression channel and completion of laminectomy under magnification can be time‐consuming, resulting in damage to the posterior bony structure and paraspinal muscles, as well as significant bleeding.^5^ In contrast, experienced surgeons may find the retroperitoneal anatomic corridor to be a more convenient and less invasive approach, preserving the integrity of the posterior bony structure and paraspinal muscles while minimizing the risk of bleeding. Therefore, based on our previous research,^14^ we innovated the microscopic ventral neural decompression technology, culminating in the development of a novel rotary distractor system called anterior lumbar discectomy and fusion (ALDF). This system introduces a unique oblique approach to direct ventral neural decompression, incorporating a set of specially designed auxiliary instruments to enhance the procedure's efficacy. At the same time, ALDF compensates for the technical shortcomings of microscope‐assisted ALIF and endoscope‐assisted OLIF. Yet, medium‐term curative effects of ALDF surgery and differences in direct ventral neural decompression between ALDF and MIS‐TLIF remain largely unknown. Therefore, the purpose of this study is: (i) to describe the meticulous and standardized technical notes of the ALDF procedure; (ii) to evaluate the clinical and radiological outcomes of the ALDF and MIS‐TLIF procedures; and (iii) to offer novel insights and strategies for the clinical treatment of LDDs that require ventral neural direct decompression.

## Materials and Methods

### Inclusion and Exclusion Criteria

The inclusion criteria were as follows: (i) single‐segment LDD including stenosis, spondylolisthesis, and lumbar instability; (ii) patients with resting radicular pain, MRI/CT confirmed extruded or sequestered disc; (iii) failure of conservative therapy (>6 months); and (iv) intervention: single spine surgery, ALDF or MIS‐TLIF.

Patients with the following conditions were excluded: (i) calcified disc herniation, stenosis due to ligamentum flavum hypertrophy or bony spurs; (ii) high‐grade spondylolisthesis; (iii) severe osteoporosis; and (4iv) follow‐up period was <3 years.

ALDF or MIS‐TLIF was chosen based on the consensus between the surgeon in charge of all surgeries and the patients. All operations were performed by a single surgical team with over 10 years of experience in minimally invasive surgery.

### ALDF Procedures

#### Anesthesia and Position

Patients were placed in a right lateral decubitus position on a radiolucent table. The right hip was slightly flexed, and the left lower extremity was extended to move away from the vascular structures and reduce the psoas volume. Fluoroscopy marked the intervertebral space and adjacent vertebral body patterns on the skin.

#### Approach and Exposure

A 4 cm skin incision was made at the left abdominal wall parallel to the obliquus externus abdominis, and the subcutaneous and fascial tissues were incised using microscopic vision. The three muscle layers of the abdominal wall were split using a blunt finger in the muscle fibers' direction to access the retroperitoneal space. The retroperitoneal fat was mobilized anteriorly with the finger to palpate the gap between the anterior border of the psoas muscle and the abdominal aorta. Under microscopic view, the retroperitoneal fat and the fascia on the surface of the psoas major, together with the ureter, were retracted anteriorly with a traditional S‐shaped retractor, and the psoas major was retracted posteriorly with an L‐shaped retractor. The anterior longitudinal ligament, left border, and lateral disc region should be visible.

#### Establish Working Channel

**FIGURE 1:**
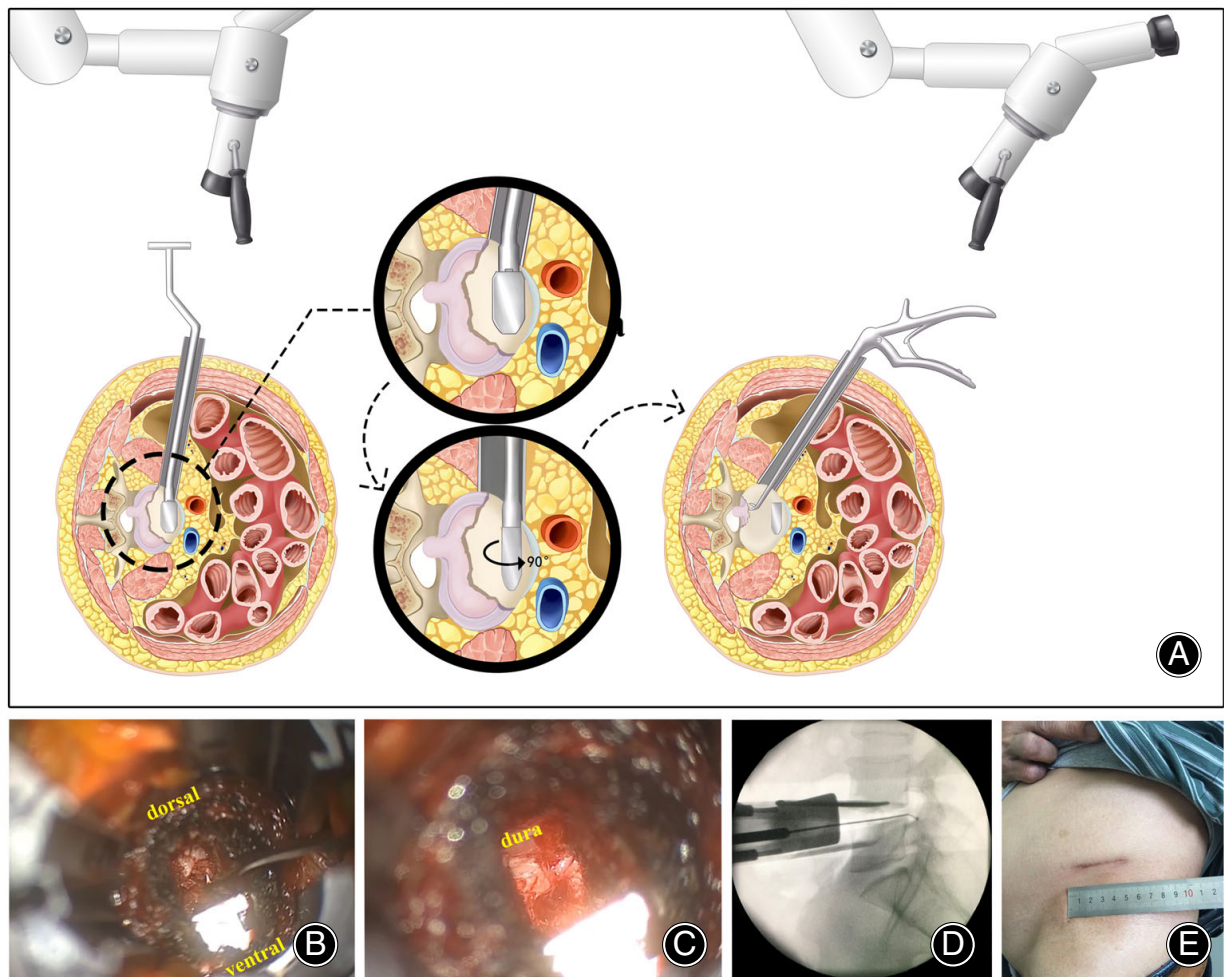
Illustration of ALDF setup and discectomy. (A) The diagram shows the specially designed rotary distractor system, the disc fragment was carefully removed. (B) The herniated disc fragments were removed intraoperatively. (C) The dural sac pulse suggested sufficient direct decompression. (D) Fluoroscopy demonstrated that the ball tip probe had reached the appropriate decompression position. (E) Incision image after ALDF surgery.

The posterior L‐shaped retractor was repositioned under the psoas muscle, a Kirschner wire was inserted into the disc space under direct vision, and the level of the correct intervertebral disc was verified by fluoroscopy with lateral images. A retractor system (Oracle cage system, DePuy Synthes, Raynham, MA, USA) was established. Although 9–11 cm retractor blades were suitable in most situations, 13 cm blades were used intraoperatively in three obese individuals. In general, the more anterior the incision, the shorter the access. Under the microscopic vision, a portion of the disk annulus was removed 2–2.5 cm posteriorly from the left edge of the anterior longitudinal ligament. A specially designed rotary distractor (WEIGO, Weihai City, China) was inserted into the intervertebral space against the anterior longitudinal ligament to ensure adequate decompression. It rotated 90° clockwise or anti‐clockwise, and the distractor module was left to maintain extended status. The microscope or operation table was tilted to obtain direct visual access to the posterior intervertebral space. In patients with left‐sided radicular symptoms, the tilt angle was much more significant (Figure 1A).

#### Discectomy and Ventral Neural Direct Decompression

A long‐handled angle curette was employed to carefully scrape the intervertebral disc tissue located at the posterior border of the intervertebral space. To quickly find the nucleus pulposus protruding into the spinal canal, a nerve hook was utilized to investigate any breaches in the annulus fibrosus. The Kerrison rongeur was then used to enlarge the breach, and the disc fragment was extracted using a hook or nucleus pulposus forceps (Figure 1B, Video S1). In our experience, the following four aspects can be utilized during ALDF surgery to determine whether the direct decompression is sufficient: (i) the prolapsed nucleus pulposus removed from within annulus fibrosus hernia sac or the area outside the breach exhibits a similar size and morphology as depicted in the radiographic images; (ii) the nerve hook reached the surface of the dural sac, the absence of compression at the posterior margin of the vertebral body and the presence of excellent dural sac pulsation (Figure 1C); (iii) fluoroscopic evidence demonstrated that the ball tip probe had reached the appropriate decompression position and that no disc material was present (Figure 1D); and (iv) if necessary, neurophysiological monitoring can sensitively detect the corresponding electromyographic response of the left or right nerve root, and provide information for judging the decompression position.

#### Fusion and Internal Fixation

**FIGURE 2:**
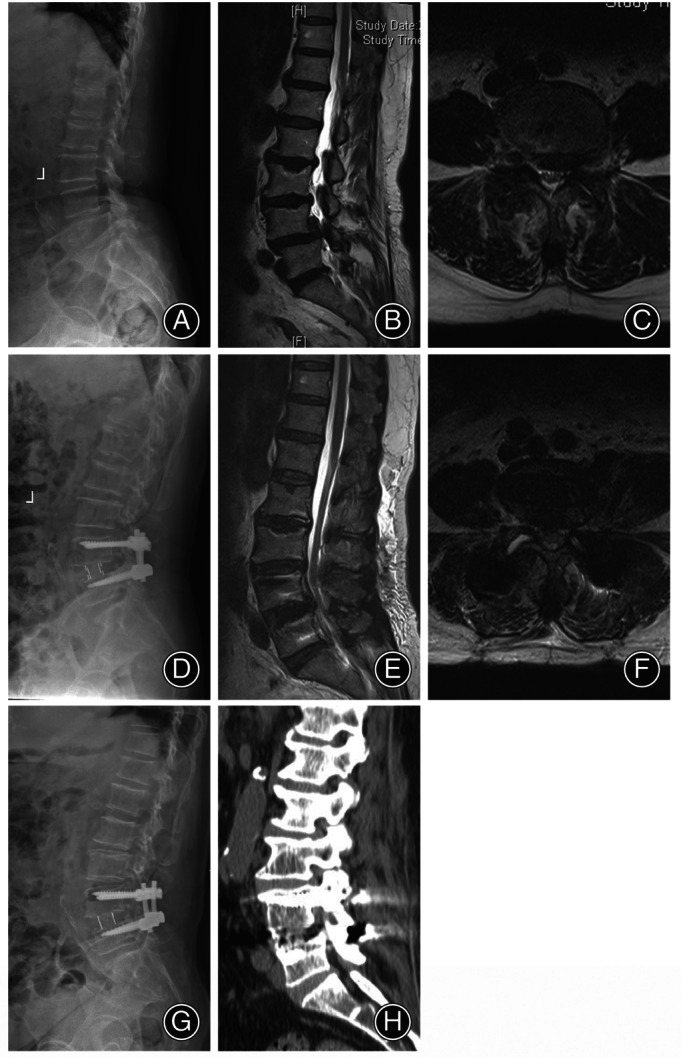
59‐year‐old female, chronic low back pain with left lower extremity radiating pain treated by ALDF. (A) Lateral radiograph shows grade II degenerative spondylolisthesis. (B, C) The sagittal and axial MRI images demonstrate central canal and left lateral recess stenosis with sequestrated disc at the L4/5 level. (D–F) X‐ray film shows excellent restoration of disc height and pedicle screw position, and MRI images show complete decompression and recovery of the cross‐sectional area 6 months after surgery. (G, H) Three‐year postoperative X‐ray and CT images show the achievement of interbody fusion.

After direct decompression was completed, a rasp was used to remove the superficial cartilaginous layers of the endplates and expose the bleeding bone. Before the cage was inserted, the operation table or channel needed to be adjusted to the standard right lateral position. Fluoroscopy ultimately verified the implant position, and the wound was closed. Subsequently, the patient was turned to the prone position, and extra posterior fixation was performed using percutaneous pedicle screw systems. A typical case in the ALDF group was shown in Figure 2.

### MIS‐TLIF Procedures

**FIGURE 3:**
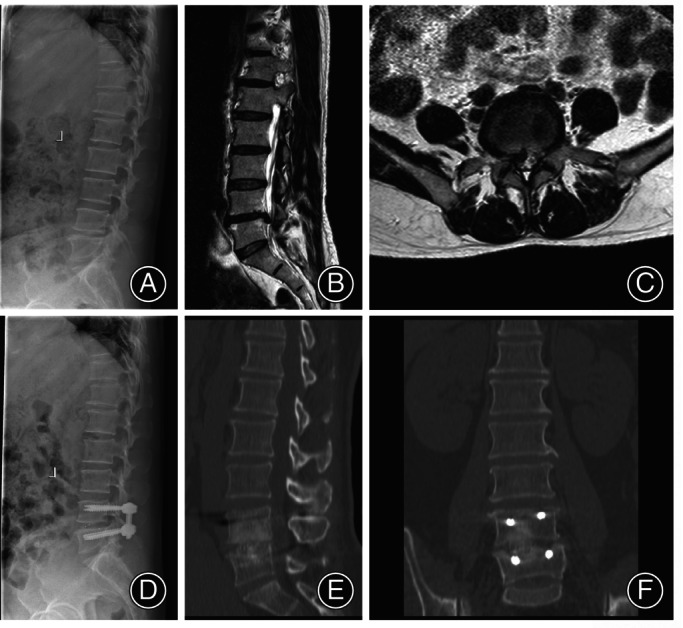
51‐year‐old female, chronic low back pain with right lower extremity radiating pain treated by MIS‐TLIF. (A–C) Lateral radiograph, the sagittal and axial MRI images demonstrate central canal and right lateral recess stenosis with the sequestered disc at the L4/5 level. (D) Six months after surgery, the X‐ray film shows excellent restoration of disc height and pedicle screw position. (E, F) Three‐year postoperative CT image showing interbody fusion has been achieved.

In the MIS‐TLIF group, the patient was placed in a prone position after general anesthesia, and the target segment was identified by fluoroscopy. A small paramedian incision was made, and a nonexpendable tubular retractor was placed *via* the Wiltse approach to reach the level of interest. A high‐speed drill was used to perform the facetectomy and decompression; the side with lower limb symptoms was defined as the decompression side according to the preoperative clinical features. After complete discectomy, direct nerve decompression, and endplate preparation, a crescent polyetheretherketone (PEEK) cage (Concorde Bullet lumbar interbody system, DePuy Spine, Raynham, MA USA) was malleted into the disc space. Lastly, bilateral pedicle screws were performed percutaneously, and the entire procedure was conducted under a surgical microscope. A typical case in the MIS‐TLIF group was shown in Figure 3.

### Outcomes Assessment

Preoperative and postoperative related radiographic parameters, including disc height (DH), segmental disk angle (SDA), lumbar lordosis (LL), and cross‐sectional area (CSA), were evaluated at baseline and 6 months, 1 year, and once a year after surgery. Detailed measurement criteria of DH, SDA, and LL were referenced from ta previous study.^13^ On axial cut T2‐weighted MRI, the CSA of the spinal canal was evaluated at the facet joint level and calculated using IMAGE J analysis software. The criterion for solid fusion was the presence of bridging trabecular bone connecting formation between the endplates on three‐dimensional CT.^15^ Three authors assessed all images individually, and the final parameter values were derived from the mean.

All patients' demographics were collected, including gender, age, body mass index, bone marrow density, preoperative diagnosis, radiculopathy, and duration of follow‐ups. Surgical data included estimated blood loss, operation time, cage height, time of getting out of bed, and complications. Clinical outcomes included scores of the visual analog scale (VAS) scores for back pain and leg pain (VAS‐back, VAS‐leg), Oswestry disability index (ODI), 36‐item short‐form health survey mental component summary (SF‐36 MCS), and physical component summary (SF‐36 PCS). Follow‐up records were acquired *via* medical records and regular outpatient visits.

### Statistical Analysis

Statistical analysis of the data in this study was performed using IBM SPSS Statistics (v 23.0; SPSS Inc., Chicago, IL, USA) statistical software. Continuous variables were reported as mean ± standard deviation, paired *t*‐tests and independent *t*‐tests were used to compare differences within and between groups, respectively, and categorical variables were statistically compared using chi‐square tests. For all analyses, *p*‐values less than 0.05 were considered statistically significant.

## Results

A total of 95 patients were analyzed, with 47 patients (20 left and 27 right) treated by ALDF and 48 patients (23 left and 25 right) treated by MIS‐TLIF. The ALDF group demonstrated less estimated blood loss (*p* < 0.001), more considerable cage height (*p* < 0.05), and shorter time of getting out of bed (*p* < 0.001) than the MIS‐TLIF group (Table 1).

**TABLE 1:** Comparison of demographic data between the two groups.

Variable	ALDF	MIS‐TLIF	t/χ^2^	*p* value
Sex (male: female)	21:26	20:28	0.088	0.767
Age (y)	61.4 ± 10.9	59.9 ± 11.7	0.374	0.383
BMI (kg/m^2^)	23.4 ± 2.9	23.8 ± 3.3	−0.653	0.359
BMD (T‐score)	−2.0 ± 0.9	−1.8 ± 1.2	−0.913	0.188
Preoperative diagnosis			3.882	0.144
Degenerative stenosis	18	21		
Degenerative spondylolisthesis	12	18		
Segmental instability	17	9		
Operation segment (%)			0.401	0.740
L3/4	4 (8.5)	6 (12.5)		
L4/5	43 (91.5)	42 (87.5)		
Radiculopathy (%)			0.276	0.600
Left	20 (42.6)	23 (47.9)		
Right	27 (57.4)	25 (52.1)		
Estimated blood loss (ml)	52.1 ± 10.7	164.3 ± 32.6	−22.425	0.000*
Operation time (min)	162.7 ± 46.2	171.4 ± 50.1	−0.927	0.178
Cage height (mm)	13.4 ± 1.5	10.8 ± 1.8	7.638	0.000*
Time of getting out of bed (h)	20.2 ± 6.5	43.3 ± 7.1	−16.526	0.000*
Follow‐up months (m)	44.1 ± 6.7	45.2 ± 5.4	−0.807	0.297

Abbreviations: BMD, bone mineral density; BMI, body mass index.

* Statistical significance between two groups.

### Radiographic Outcomes

The DH, SDA, and CSA considerably increased at 6 months postoperatively except for LL and were well preserved at 3 years postoperatively in two groups compared with the preoperative data (all *p* < 0.05). In terms of group comparisons, the ALDF group had significantly better DH and SDA than the MIS‐TLIF group at 6 months, 1 year, and 3 years (*p* < 0.05). The fusion rates of the ALDF group were 85.1% at 6 months postoperatively, significantly higher than the 66.7% of the MIS‐TLIF group (*p* < 0.05) (Table 2).

**TABLE 2:** The radiographic outcomes comparison between the two groups.

Variable	ALDF	MIS‐TLIF	t/χ^2^	*p* value
DH (mm)				
Baseline	7.3 ± 2.8	7.5 ± 2.7	−0.352	0.771
6 mos	13.1 ± 1.7*	10.2 ± 1.5*	8.811	0.000**
1 yr	12.6 ± 1.9*	9.5 ± 1.8*	8.162	0.000**
3 yrs	11.8 ± 2.4*	9.2 ± 2.1*	5.623	0.000**
SDA (°)				
Baseline	9.3 ± 1.9	9.6 ± 2.3	−0.633	0.405
6 mos	12.2 ± 3.3*	15.3 ± 4.8*	−4.662	0.000**
1 yr	11.9 ± 3.7*	13.1 ± 4.2*	−4.476	0.000**
3 yrs	10.2 ± 3.4*	11.8 ± 3.6*	−4.012	0.000**
LL (°)				
Baseline	40.3 ± 11.9	40.1 ± 12.7	0.079	0.754
6 mos	42.1 ± 12.4	40.2 ± 11.5	0.678	0.282
1 yr	41.8 ± 13.6	39.7 ± 12.3	0.489	0.356
3 yrs	39.4 ± 12.9	38.8 ± 11.7	0.208	0.722
CSA (mm^2^)				
Baseline	58.0 ± 19.1	59.3 ± 19.4	−0.329	0.737
6 mos	122.2 ± 13.8*	123.0 ± 13.5*	−0.286	0.784
1 yr	125.9 ± 12.9*	127.2 ± 12.6*	−0.449	0.583
3 yrs	125.7 ± 12.8*	126.8 ± 12.7*	−0.420	0.624
Fusion rate (n)				
6 mos	85.1% (40)	66.7% (32)	4.401	0.036**
1 yr	89.4% (42)	83.3% (40)	0.731	0.393
3 yrs	95.7% (45)	91.7% (44)	0.667	0.414

Abbreviations: CSA cross‐sectional area; DH disc height; LL lumbar lordosis; SDA segmental disk angle.

* Significantly different from baseline data.

** Statistical significance between two groups.

### Clinical Outcomes

The average VAS‐back scores of the ALDF group significantly decreased from 6.3 ± 1.3 preoperatively to 1.7 ± 1.3 at 6 months postoperatively and was maintained at 3 years postoperatively (*p* < 0.05), with a remission rate of 82.9%. In the MIS‐TLIF group, the average VAS‐back scores dropped considerably from 6.5 ± 1.6 preoperatively to 2.9 ± 1.5 at 6 months postoperatively and 2.1 ± 1.1 at 3 years postoperatively (*p* < 0.05), with a remission rate of 68.6%. The VAS‐back score and its remission rate were significantly improved in the ALDF group compared to the MIS‐TLIF group (*p* < 0.05). The average VAS‐leg score significantly decreased at 3 years postoperatively in the ALDF group and MIS‐TLIF group (*p* < 0.05). Moreover, patients in both groups improved significantly in the SF‐36 PCS and SF‐36 MCS scores after surgery until 3 years of follow‐up (all *p* < 0.05). However, there was no significant difference between the two groups (all *p* > 0.05) (Table 3).

**TABLE 3:** The clinical outcomes comparison between the two groups.

Variable	ALDF	MIS‐TLIF	t	*p* value
VAS‐back				
Baseline	6.3 ± 1.3	6.5 ± 1.6	−0.580	0.408
6 mos	1.7 ± 1.3*	2.9 ± 1.5*	−8.163	0.000**
1 yr	1.4 ± 0.6*	2.5 ± 1.2*	−7.885	0.000**
3 yrs	1.2 ± 0.5*	2.1 ± 1.1*	−6.156	0.000**
VAS‐leg				
Baseline	7.3 ± 1.2	7.4 ± 1.1	−0.424	0.636
6 mos	1.4 ± 1.1*	1.6 ± 0.9*	−0.906	0.252
1 yr	0.9 ± 0.7*	1.0 ± 0.8*	−0.694	0.324
3 yrs	0.7 ± 0.6*	0.8 ± 0.6*	−0.691	0.331
ODI				
Baseline	64.6 ± 12.5	66.1 ± 12.7	−0.652	0.385
6 mos	16.3 ± 2.3*	17.5 ± 2.8*	−2.269	0.112
1 yr	11.3 ± 2.8*	11.9 ± 3.0*	−0.885	0.264
3 yrs	7.9 ± 2.0*	8.3 ± 2.2*	−0.497	0.426
SF‐36 PCS				
Baseline	27.7 ± 6.2	28.9 ± 6.7	−0.891	0.284
6 mos	48.3 ± 9.4*	46.9 ± 9.2*	0.834	0.158
1 yr	57.8 ± 10.7*	56.6 ± 10.2*	0.568	0.326
3 yrs	61.6 ± 8.8*	60.7 ± 9.0*	0.223	0.615
SF‐36 MCS				
Baseline	34.4 ± 8.0	35.9 ± 8.4	−0.882	0.291
6 mos	67.6 ± 12.1*	65.9 ± 13.3*	−1.352	0.132
1 yr	71.7 ± 11.7*	70.6 ± 12.4*	0.772	0.259
3 yrs	73.8 ± 9.5*	73.1 ± 10.9*	0.154	0.738

Abbreviations: MCS, mental component summary scores; PCS, physical component summary scores.

* Significantly different from baseline data,

** Statistical significance between two groups.

### Complications

Significant complications are listed in Table 4, with all accepted immediate and effective treatments. In the complication rates, ALDF was slightly higher than the MIS‐TLIF group (10.6% *vs*. 8.4%), and there was no significant difference between the two groups (*p* > 0.05).

**TABLE 4:** Summary of treatments for complications of ALDF and MIS‐TLIF.

Variable	n (%)	Treatment and prognosis
ALDF	5 (10.6)	
Temporary psoas injury	1 (2.1)	Symptoms were resolved 3 days after surgery with neurotrophic treatment
Cage subsidence	2 (4.3)	Thoracic lumbar braces were used for protection until bony fusion
Lumbar plexus injury	1 (2.1)	Symptoms were resolved 3 days after surgery with neurotrophic treatment
Genitofemoral nerve injury	1 (2.1)	Symptoms were resolved 6 days after surgery with neurotrophic treatment
MIS‐TLIF	4 (8.4)	
Dural tear	1 (2.1)	The intraoperative dural was repaired, and no postoperative cerebrospinal fluid leaking occurred.
Cage subsidence	2 (4.2)	Thoracic lumbar braces were used for protection until bony fusion
Asymptomatic ASD	1 (2.1)	Follow‐up observation regularly

Abbreviation: ASD, adjacent segment disease.

## Discussion

Due to OLIF, microscope‐assisted ALIF, and spinal endoscope‐assisted OLIF are deemed inappropriate for instances involving large prolapsed discs located in the left lateral recess. In this study, we show a novel surgical method called ALDF and address the limitations of preceding methodologies. Upon conducting a follow‐up of at least 3 years, ALDF demonstrated superior clinical results compared to MIS‐TLIF in LDDs with huge disc herniation, regardless of left or right radiculopathy.

### Technical Flaws of Microscope‐assisted ALIF and Spinal Endoscope‐Assisted OLIF

OLIF is a minimally invasive spine procedure providing indirect decompression to neural elements by decreasing intervertebral disc bulging, unbuckling the ligamentum flavum, expanding the spinal canal, and restoring disc and foraminal height.^7^, ^16^, ^17^ However, LDDs with static extruded or sequestered discs may not have good decompression effects, which is also considered a contraindication to OLIF.^6^, ^9^, ^10^, ^13^, ^18^ More sufficient and direct decompression or supplementary posterior decompressive procedure after indirect decompression may be necessary.^19^, ^20^ Additionally, spine surgeons try to use microscope‐assisted ALIF and spinal endoscope‐assisted OLIF to complete ventral neural decompression.^6^, ^8^, ^9^, ^10^, ^11^, ^18^, ^20^, ^21^ However, the anatomical complexity of the anterior approach and the limited surgical space due to the loss of intervertebral height may make the clinical application of these two techniques more challenging. In this study, ALDF remedied the previously reported technical flaws. The surgical procedure and decompression principle are entirely distinct from traditional OLIF surgery and compensate for the technical shortcomings of microscope‐assisted ALIF and endoscope‐assisted OLIF.

### ALDF Promoted Rapid Rehabilitation

A meta‐analysis showed that the average operation time for OLIF combined with posterior internal fixation for single lesions was 54.5 min, but for MIS‐TLIF it was 166.4 min.^2^ Our results showed that ALDF required longer surgery time than OLIF, and surgical procedure time in MIS‐TLIF generally agreed with other studies. The main reason for this difference is that ALDF requires extra time to complete discectomy and remove the prolapsed nucleus pulposus within the annulus fibrosus hernia sac or the area outside the breach. For both direct nerve decompression procedures, we observed that ALDF required a slightly shorter operative time than MIS‐TLIF, although this was not statistically different. Additionally, we noticed that intraoperative blood loss in the ALDF group was significantly less than that in the MIS‐TLIF group. We considered two reasons. First, the ALDF technique was performed through the oblique lateral corridor and accessed by blunt dissection without destroying the posterior column structures. Second, under a microscope, the surgeon could carefully and precisely remove the disc materials without causing endplate damage, hemorrhage, or collapse. For the MIS‐TLIF technique, damaging the lumbar paraspinal muscles and facet capsule, to some extent, increased the possibility of intraoperative bleeding.^22^ In our study, patients in the ALDF group had a shorter time getting out of bed than the MIS‐TLIF group. This might be the absence of integrity of the facet joints, and hard‐to‐perceive screw fretting on the MIS‐TLIF decompression side due to osteoporosis. In contrast, the ALDF preserved the integrity of the facet joint capsule, provided superior spinal stability, facilitated early ambulation, and promoted rapid rehabilitation.

### Comparison of Imaging Parameters

Radiographically, ALDF resulted in better DH and SDA improvement than MIS‐TLIF, whereas LL did not. Several reasons could be associated with this finding. First, the wide cage was mounted on the rigid epiphyseal ring surrounding the vertebral body in ALDF, as opposed to the relatively weak bone cortex in the central depression of the endplate, thereby effectively preventing DH loss and cage subsidence. Second, the cage radian from front to back is 6° or 12° in ALDF but 0° in MIS‐TLIF, which was theoretically beneficial to restoring SDA. Third, there is a slight improvement in LL with single‐level fusion, whereas multi‐level ALDF surgery may achieve statistically significant improvement. Furthermore, in our series, the expansion rate of CSA was comparable between MIS‐TLIF and ALDF at 3 years postoperatively (110.7% and 107.4%, *p* > 0.05), which indicated equivalent effects were produced by the two surgical procedures on the direct decompression of the spinal canal.

### ALDF Achieves Satisfactory Medium‐term Clinical Efficacy

Our results showed that both ALDF and MIS‐TLIF are beneficial in improving VAS, ODI, and SF‐36 scores at both short‐ and medium‐term follow‐ups. In parallel, we found that the VAS‐back score following ALDF was lower, and the remission rate of low back pain was higher than those of MIS‐TLIF at 3 years follow‐up (82.9% and 68.6%, *p* < 0.05). This could be because ALDF maintained better DH and SDA at the last follow‐up. In the meantime, we observed a higher fusion rate based on CT in the ALDF group at 6 months, which may be due to the larger contact area of the bone graft, although the 3‐year fusion rates of the two groups were comparable. Historically, oblique lateral approach surgery had a high incidence of complications, and previous studies reported that the incidence ranged from 15.6% to 48.4%.^4^, ^19^, ^23^, ^24^ Yet, our data are lower than the ranges described in the literature. Notably, dura tears and cerebrospinal fluid leakage, which can occur with the other anterior approach, were not observed in our ALDF patients. These differences are most likely attributed to the microscope offering a more precise surgical view for identifying the ureter, segmental vessel, sympathetic chain, cartilage endplate, and dural sac. Although direct ventral neural decompression is more difficult in left radiculopathy,^6^, ^11^, ^18^ our study reveals that ALDF can achieve satisfactory medium‐term efficacy in both left and right radiculopathy through subtly tilting the operating table or microscope. There is no denying the fact that the implantation of channels of ALDF operation needs to pull the psoas major muscle to the rear and place retractor blades. Ensuring that all operations are under clear direct vision and without any entrapment of soft tissue in the channel is crucial to prevent nerve damage. Nevertheless, we still observed two cases of damage to the lumbar plexus and genitofemoral nerve. Fortunately, the symptoms of nerve injury in these two patients rapidly recovered under neurotrophic treatment. Overall, ALDF provides a new surgical scheme for the treatment of LDDs with huge disc herniation and retains the advantage of lateral lumbar fusion without destroying the structure of the posterior ligament complex, as well as the advantage of direct decompression of the spinal canal, which has a certain clinical significance.

### Limitations and Prospects

First, there was selection bias because patients were not randomly assigned to the ALDF or MIS‐TLIF group and were only assigned to one of the treatments after being apprised of the procedure in detail. Second, this is a retrospective, single‐center study that is susceptible to the inherent limitations of retrospective analyses. Multi‐center, large‐sample, and prospective clinical studies are required to validate these conclusions further. Third, the ALDF procedure requires a comprehensive knowledge of the anatomy of the blood vessels around the spine and the operation requires a certain learning curve.

## Conclusions

ALDF demonstrated good medium‐term outcomes in LDDs with huge disc herniation, regardless of left or right radiculopathy, and showed advantages in improving radiographic outcomes, increasing the remission rate of low back pain, and promoting rapid recovery. Although ALDF presents slightly higher complication rates compared to MIS‐TLIF, it does not adversely affect clinical outcomes. In the future, ALDF may be the preferred surgical treatment for suitable LDDs patients.

## Ethics Statement

This study was performed in line with the principles of the Declaration of Helsinki. Approval was granted by the Ethics Committee of Fujian Provincial Hospital (No. K2021‐11‐003).

## Conflict of Interest Statement

The authors report no conflict of interest concerning the materials or methods used in this study or the findings specified in this paper.

## Author Contributions

All authors contributed to the study's conception and design. Data collection and analysis were performed by Chao Song, Zhibo Deng, Hanhao Dai, and Wu Zheng. The first draft of the manuscript was written by Chao Song and all authors commented on previous versions of the manuscript. All authors read and approved the final manuscript.

## Supporting information


**Video S1.** Supplemental file.
